# The effect of Gegen Qinlian Decoction on clinical prognosis and islet function for type 2 diabetic mellitus: A protocol for systematic review and meta-analysis

**DOI:** 10.1097/MD.0000000000024210

**Published:** 2021-02-05

**Authors:** Yuhang Ren, Peiyu Xiong, Chun Zhong, Peixu Zhang, Bo Jia

**Affiliations:** College of Basic medicine, Chengdu University of Traditional Chinese Medicine, Chengdu, P.R. China.

**Keywords:** clinical prognosis, gegen qinlian decoction, islet function, protocol, systematic review, type 2 diabetic mellitus

## Abstract

**Background:**

With the development of social economy, people's lives are improving day by day. Chronic diseases represented by diabetes have gradually entered people's field of vision. At present, about 415 million people in the world suffer from diabetes, of which more than 90% are Type 2 diabetic mellitus (T2DM), which causes severe physical and mental pain to patients and their families, and also imposes a huge burden on the health care system. Animal experiments and clinical studies both show that Gegen Qinlian Decoction (GQD) cannot only reduce the blood glucose of T2DM, but also improve the islet function of patients, reduce the insulin resistance index and insulin secretion index, and have no adverse reactions. Therefore, we designed this protocol to evaluate the effect of GQD on clinical Prognosis and islet function for T2DM.

**Methods:**

This review was conducted from January 1, 2000 to October 1, 2020, sourced from the Cochrane Library, Pubmed, Excerpt Medica Database, Science Direct, World Health Organization, International Clinical Trials Registration Platform, Web of Science, Chinese Biomedical Literature, the China National Knowledge Infrastructure Database, Wanfang Database, Chinese Scientific Journal Database. In this study clinical randomized controlled trial is used and we set inclusion criteria and exclusion criteria for screening. The primary outcomes include Fasting plasma glucose,2 h plasma glucose, Hemoglobin A1c, fasting plasma insulin, insulin resistance index and insulin secretion index. Review Manager 5.3 software will be used for data analysis.

**Results:**

This study will provide the systematic evidence of the effect of GQD on Clinical Prognosis and islet function for T2DM.

**Conclusion:**

The findings of this meta-analysis will provide evidence to evaluate the effect of GQD on Clinical Prognosis and islet function for type 2 diabetic mellitus.

**INPLASY registration number:**

INPLASY2020110083.

## 1 Introduction

With the development of social economy, people's lives are improving day by day. Chronic diseases represented by diabetes have gradually entered people's field of vision. The latest data released by the International Diabetes Federation IDF in 2019 (9th edition) shows that in 2019, approximately 463 million adults aged 20 to 79 worldwide is suffering from diabetes, accounting for 1/11 of the total population; International Diabetes Federation predicts that in 2030 The number of diabetes patients worldwide could rise to 578.4 million.^[1]^ T2DM (type 2 diabetic mellitus) patients account for 90% of the diabetic patients. T2DM has become one of the important chronic diseases that damage the health of the people of the world. It has brought serious health economic burdens to the country, society and families, and is a major and urgent public health issue that need to be solved. At this stage, the understanding of the etiology and pathological mechanism of diabetes is still insufficient. It is currently believed that insulin resistance is the initiating factor leading to increased blood glucose, so improving insulin resistance has become a key step to prevent the occurrence and development of diabetes.^[2]^

Patients with T2DM usually have pathological factors of insulin resistance, it leads to impaired insulin function, insufficient insulin secretion, and long-term hyperglycemia. High glucose toxicity further induces inflammation and oxidative stress, which ultimately induces serious complications such as diabetic nephropathy and cardiovascular disease.^[3–7]^ The damage of pancreatic β-cells and their function have been run through from the initiation of diabetes. The protection of pancreatic islet function is the core treatment of diabetes.^[8]^ At present, Western medicine treatment methods mostly focus on the control of patients’ blood glucose and improvement of symptoms, but diabetes cannot be cured, and there often are side effects of varying degrees.^[9]^

Traditional Chinese medicine now plays an important role in the treatment of chronic diseases such as diabetes and is gradually being recognized worldwide. Gegen Qinlian Decoction is derived from Shanghanlun wrote by Zhang Zhongjing (150-219CE). It can be used for the treatment of diabetes. The medicine composition includes Gegen (Puerariae Lobatae Radix), Huangqin (Scutellaria Baicalensis), Huanglian (Coptis), and Gancao (Glycyrrhiza Uralensis), Animal experiments show that GQD can inhibit miR-146b, up-regulate SIRT1 level, reduce FoxO1 acetylation, and improve liver insulin resistance.^[10]^ Clinical studies also show that GQD cannot only reduce the blood glucose of T2DM, but also improve the islet function of patients, reduce the insulin resistance index and insulin secretion index, and have no adverse reactions.^[11]^ There is no systematic review and meta-analysis of the effects of GQD on the clinical prognosis and islet function of T2DM. Therefore, we wrote this protocol for systematic review and meta-analysis to provide stronger evidence to guide clinical practice.

## 2 Methods

### 2.1 Protocol registration

This protocol had been registered with the International Platform of Registered Systematic Review and Meta-Analysis Protocols (INPLASY), the registration number is INPLASY2020110083, and the doi number is 10.37766/inplasy2020.11.0083. This study will be followed the guidelines of Preferred Reporting Items for Systematic Review and Meta-Analysis Protocols (PRISMA-P).^[12]^

### 2.2 Inclusion criteria

#### 2.2.1 Types of study

This study includes randomized controlled trials and observational studies: cohort prospective or retrospective, case-control and cross-sectional studies.

#### 2.2.2 Participants

The patients of T2DM (using WHO 1999 diagnostic criteria). These types of patients will not be included: Patients with severe impairment of islet function; patients with severe diabetic complications; patients with severe liver and kidney lesions; patients with acute cardiovascular and cerebrovascular diseases; poor patient compliance; pregnant or lactating women.

#### 2.2.3 Types of intervention

According to the conventional diabetes treatment methods recommended by the ADA guidelines,^[13]^ including diet, exercise, and hypoglycemic and lipid lowering treatments combined with GQD or modified GQD.

### 2.3 Type of outcomes

#### 2.3.1 Primary outcomes

The primary outcomes include fasting plasma glucose,2 h plasma glucose, hemoglobin A1c, fasting plasma insulin, insulin resistance index and insulin secretion index.

#### 2.3.2 Secondary outcomes

The secondary outcomes include clinical efficacy and adverse reactions. The clinical efficacy refers to the guiding principles for clinical research of new Chinese medicines.^[14]^

Significantly effective: symptoms improved significantly more than 70%, the fasting blood glucose is less than 7.0 mmol/L, and the blood glucose 2 hours after a meal is less than 8.3 mmol/L; effective: symptoms reduced by 30% to 70%, and the fasting blood glucose is less than 7.0∼9.0 mmol/L. In the meantime, the blood glucose in 2 hours after meal is between 8.3∼10.5 mmol/L; Ineffective: Symptom improvement is less than 30% or no improvement, or even worse, fasting blood glucose is higher than 9.0 mmol/L, and blood glucose 2 hours after meal is higher than 10.5 mmol/L. Total effective rate = (significantly effect + effective)/total number of cases × 100%. Adverse reactions: nausea, abdominal pain, dyspepsia, and dizziness were used as adverse reaction indicators.^[11]^

### 2.4 Search Strategy

This review was conducted from January 1, 2000 to October 1, 2020, sourced from the Cochrane Library, Pubmed, EMBASE, Science Direct, World Health Organization (WHO), International Clinical Trial Registration Platform, Web of Science,Chinese Biomedical Literature, the China National Knowledge Infrastructure Database, Wanfang, Database,Chinese Scientific Journal Database, No limitation on language or publication types restriction will be applied. The following key terms are used: Gegen Qinlian Decoction, clinical prognosis, islet function, blood glucose. We will change the retrieval strategy according to the different databases. The process of search is shown in Table 1.

**Table 1:** Search strategy for the PubMed database.

#1 Type 2 Diabetic Mellitus [Title/Abstract]
#2 Type 2 Diabetes [Title/Abstract]
#3 #1OR#2
#4 Gegen Qinlian Decoction [Title/Abstract]
#5 Clinical Prognosis [Title/Abstract]
#6 Islet function [Title/Abstract]
#7 #3 AND#4 AND [#5 OR#6]
#8 Blood Glucose [Title/Abstract]
#9 Blood Sugar [Title/Abstract]
#10 #8OR#9
#11 Insulin Resistance [Title/Abstract]
#12 Insulin Sensitivity [Title/Abstract]
#13 #11 OR#12
#14 Insulin Secretion [Title/Abstract]
#15 #3 AND#10 AND [#13 OR#14]
#16 Type 2 Diabetes Mellitus [Mesh Terms]
#17 Traditional Chinese Medicine Treatment [Mesh Terms]
#18 [#3 OR#6] AND#17
#19 #7 OR#15 OR#18
#20 Clinical [Title/Abstract]
#21 Trial [Title/Abstract]
#22 #20 AND#21
#23 clinical trials as topic [Mesh Terms]
#24 clinical trial [Publication Type]
#25 random [Title/Abstract]
#26 random allocation [Mesh Terms]
#27 therapeutic use [Mesh Subheading]
#28 #22 OR#23 OR#24 OR#25 OR#26 OR#27
#29 #19 AND#28

### 2.5 Studies selection

**Figure 1:**
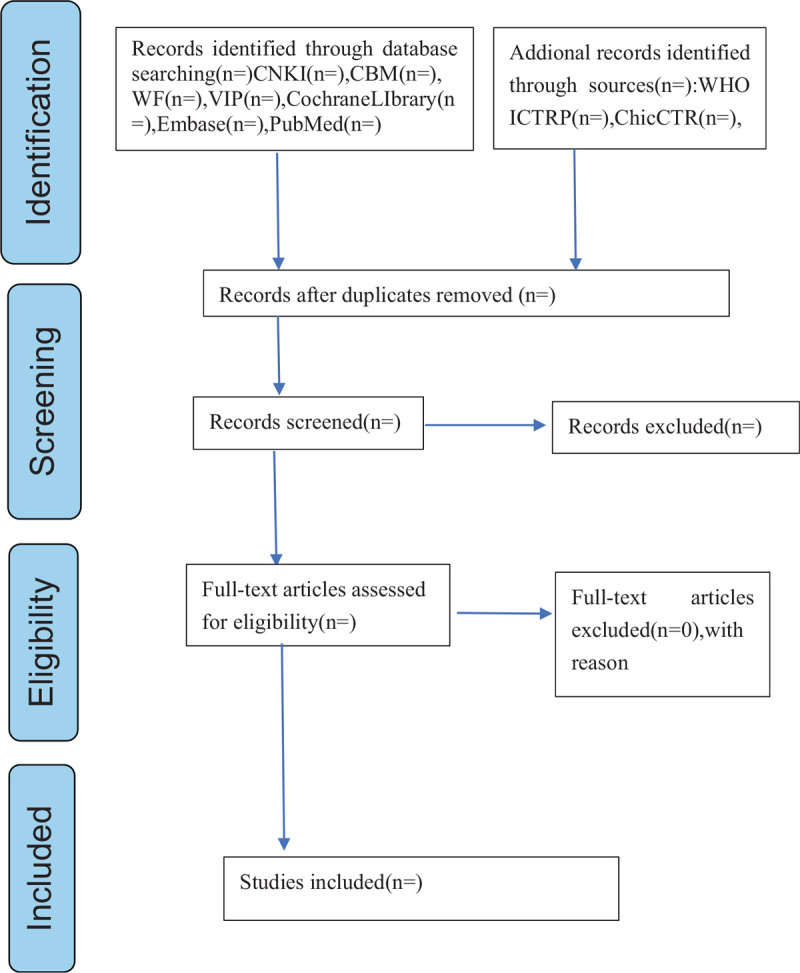
Flow diagram of the study selection process.

Research screening will be conducted independently by 2 researchers based on the inclusion criteria. All results should be imported into endnote X9, and duplicates will be deleted. The researcher will read the title and abstract, exclude irrelevant literature (the reasons for excluding the study will be written down), and then read the full text. The literature will be included in the study if it meets the inclusion criteria. If the 2 authors disagree during the selection process, we will discuss and resolve them with the third author. The process of this meta-analysis will appear in the form of a PRISMA flowchart (Fig. 1).

### 2.6 Data extraction and management

Two researchers used standardized extraction tables to extract data. The extracted data will include title, author, year, gender, age, sample size, intervention measures, results, etc. Any discrepancies will be resolved through group discussion.

### 2.7 Assessment of risk of bias

All the included studies will be assessed based on the guidelines of Cochrane Handbook for Systematic Reviews of Interventions.^[15]^ The evaluation contents include: blindness of participants and personnel, blindness of result evaluation, generation of random sequence, concealment of distribution, incomplete result data, selective result report and other sources of bias. The quality of each trial will be categorized into 3 levels: “low bias,” “high bias,” and “unclear bias.” Any disagreements between the reviewers of both parties will be analyzed by the third-party reviewer and reached an agreement.

### 2.8 Data synthesis

The risk ratio (RR) of relative risk with 95% confidence interval (CI) was used to summarize the binary results. Continuous results will be summarized by using weighted mean difference (WMD) with 95% CI. According to the research recommendations, we will use the random effects model (REM) for meta-analysis in this paper. Statistical heterogeneity will be evaluated by X^2^ And I^2^ statistical tests. When *P* ≥ .1 and I^2^ ≤ 50%, there is no significant statistical heterogeneity between the studies. On the contrary, the *P* value 50% indicates a considerable heterogeneity. When the statistical heterogeneity is acceptable (P value ≥ .1, I^2^ ≤ 50%), a meta-analysis will be conducted; otherwise, a subgroup analysis will be conducted to explore the impact of potential factors on the outcome measurement.

### 2.9 Subgroup analysis

According to different races, ages, genders, duration of treatment, and different types of GQD (intervention forms, drug formulations, dosages, etc.), subgroup analysis will be performed. If meta-analysis is not possible, we will conduct a descriptive analysis.

### 2.10 Sensitivity analysis

We will omit studies one by one to conduct sensitivity analysis to explore the impact of individual study.

### 2.11 Grading the quality of evidence

The Grading of Recommendations Assessment, Development and Evaluation (GRADE) will be used for evaluating the quality level of evidence.^[16]^

### 2.12 Ethics and dissemination

This study is a secondary study based on published clinical studies. Therefore, this study does not require ethics committee approval. According to the preferred reporting project of the Systematic Review and Meta-Analysis Protocol (PRISMA-P), the results of this research will be published in peer-reviewed scientific journals and conference papers.

## 3 Discussion

The people's living standards are improving day by day, and the changing lifestyles have led to a rapid increase in the incidence of diabetes. The prevalence of diabetes worldwide is 6.5%, among which T2DM is the main type.^[17,18]^ The incidence of T2DM is gradually turning to younger age. The proportion of early-onset patients before the age of 40 is gradually increasing. It is more difficult for adolescents to control blood glucose, and hyperlipidemia is more serious, which greatly increases the incidence of diabetes complications.^[19,20]^ Diabetes complications are complex and numerous, with high disability and fatality rates, which seriously endanger the quality of life and health of patients. Effective and reasonable intervention to control diabetes is of great significance.^[21,22]^ According to the reference guide, the current drug for the treatment of diabetes is mostly sulfonylureas, which are widely used in this field. A large amount of clinical experience has proved that sulfonylureas have good effects in reducing blood glucose and delaying complications. However, there are certain discussions about the safety and application risks of such drugs.^[23–25]^ GQD is used to treat T2DM. Animal experiments have confirmed that it can improve insulin resistance in T2DM rats.^[26]^ Clinical studies have shown that GQD can improve pancreatic islet's function and control blood glucose levels in patients.^[27]^ However, the effect of GQD on the clinical prognosis and islet function of T2DM is not known. Therefore, it is necessary to conduct a systematic review to provide objective evidence for the clinical prognosis and improvement of islet function of GQD for T2DM. The scheme will conduct a meta-analysis of existing clinical literature to provide systematic reference for clinical treatment of T2DM.

## Author contributions

**Conceptualization:** Yuhang Ren, Chun Zhong

**Data curation:** Yuhang Ren, Peiyu Xiong

**Formal analysis:** Yuhang Ren.

**Funding acquisition:** Bo Jia, Peiyu Xiong.

**Methodology:** Yuhang Ren.

**Project administration:** Bo Jia.

**Resources:** Yuhang Ren, Peixu Zhang.

**Software:** Peixu Zhang.

**Supervision:** Bo Jia.

**Writing – original draft:** Yuhang Ren.

**Writing – review & editing:** Bo Jia.
